# High-Dimensional Additive Hazards Regression for Oral Squamous Cell Carcinoma Using Microarray Data: A Comparative Study

**DOI:** 10.1155/2014/393280

**Published:** 2014-05-19

**Authors:** Omid Hamidi, Lily Tapak, Aarefeh Jafarzadeh Kohneloo, Majid Sadeghifar

**Affiliations:** ^1^Department of Science, Hamadan University of Technology, Hamedan 65155, Iran; ^2^Department of Biostatistics and Epidemiology, School of Public Health, Hamadan University of Medical Sciences, Hamadan 6517838695, Iran; ^3^Department of Epidemiology & Biostatistics, School of Public Health, Hamadan University of Medical Sciences, Hamadan, Iran; ^4^Department of Statistics, Faculty of Science, Bu-Ali Sina University, Hamadan 6517838695, Iran

## Abstract

Microarray technology results in high-dimensional and low-sample size data sets. Therefore, fitting sparse models is substantial because only a small number of influential genes can reliably be identified. A number of variable selection approaches have been proposed for high-dimensional time-to-event data based on Cox proportional hazards where censoring is present. The present study applied three sparse variable selection techniques of Lasso, smoothly clipped absolute deviation and the smooth integration of counting, and absolute deviation for gene expression survival time data using the additive risk model which is adopted when the absolute effects of multiple predictors on the hazard function are of interest. The performances of used techniques were evaluated by time dependent ROC curve and bootstrap .632+ prediction error curves. The selected genes by all methods were highly significant (*P* < 0.001). The Lasso showed maximum median of area under ROC curve over time (0.95) and smoothly clipped absolute deviation showed the lowest prediction error (0.105). It was observed that the selected genes by all methods improved the prediction of purely clinical model indicating the valuable information containing in the microarray features. So it was concluded that used approaches can satisfactorily predict survival based on selected gene expression measurements.

## 1. Introduction

Head and neck squamous cell carcinoma (HNSCC) is the sixth most common cancer worldwide that has variation across subsites (oral cavity, oropharynx, larynx, or hypopharynx) in many characteristics including age, sex, ethnicity, histologic grade, treatment modality, and prognosis [1–3]. HNSCC as the most common smoking related cancer after lung cancer is one of the most aggressive malignancies in human population [1, 3] and causes about 438,000 smoking-attributable mortalities in the world each year. The most common anatomic site of HNSCC counting for approximately 50% of all HNSCC is oral squamous cell carcinoma (OSCC) which contains two most commonly diagnosed oral premalignant lesions (OPL) including Leukoplakia and erythroplakia [3].

Although tremendous efforts were committed to early detection, prevention, and treatment during the last decades, HNSCC prognosis remains very poor with the rising incidence in developed countries and younger population [1]. Even for HNSCC diagnosed at early stages, surgery (current standard care) is a debilitating, substantially morbid procedure that leads to severe impairments in quality of life for the patients [3]. Therefore, developing new approaches including diagnosis of the disease before the cancerous stage and preventing development of invasive cancers such as HNSCC is very substantial. The HNSCC is a multistep process and genetic factors play an important role in its etiology [1]. Generally, it is well accepted that malignant tumors, on the molecular level, are disease of genes [1].

The rapid development of biotechniques during the past decade has brought in a wealth of biomedical data including DNA microarrays which can be used to measure the expression of thousands of genes in a sample of cells or to identify hundreds of thousands of single nucleotide polymorphisms for an individual at the same time [4]. This kind of information leads to high-dimensional and low-sample size (HDLSS) data sets (i.e., *p* ≫ *n* where *p* and *n* are the number of covariates and patients) which pose tremendous challenges to effective statistical inference especially for the time-to-event due to the presence of censoring and the use of much more complicated models. In this case, a number of the outcome variables are typically censored. Besides, establishing link between high-dimensional biomedical data and patient's survivals would be informative in the presence of clinical data.

In this regard, a fundamental objective is to identify a small set of genes from a huge number of features whose expression levels are significantly correlated with a given clinical outcome such as cancer. Identified genes are then often used to create a predictive model for conducting prediction of outcome for new patients [5].

Recently, several variable selection techniques based on the maximization of a penalized likelihood have been proposed for HDLSS time-to-event data under the Cox proportional hazards model. Some of the most common penalization methods are the least absolute shrinkage and selection operator (lasso) [6], smoothly clipped absolute deviation (SCAD) [7], Dantzig selector [8], LARS [9], and the smooth integration of counting and absolute deviation (SICA) penalty [10]. These techniques can yield sparse models and hence perform simultaneous variable selection and estimation. To select ideal penalty functions for model selection Fan and Li [7] advocated penalty functions giving rise to estimators with three desired properties of sparsity, unbiasedness, and continuity. This class of penalty functions has been studied by Lv and Fan [11] and Fan and Lv [12] in (generalized) linear models contexts. The reasonable performance of penalization techniques such as Lasso, SCAD, and SICA was also investigated by Lin and Lv [4].

Despite the extensive study of the proportional hazards model for survival data with high-dimensional covariates, a few authors have utilized variable selection methods based on additive risk model which may provide more insights beyond the proportional hazards model analysis [4, 13]. Additive models assume that the covariate effects under consideration contribute additively to the conditional hazard [13].

The aim of the present study was to compare three sparse variable selection methods of Lasso, SICA, and SCAD for high-dimensional low-sample size data using an additive hazard approach to predict survival time in patients with OSCC and to determine the influential genes on survival time.

## 2. Methods

### 2.1. Data Source

The proposed techniques are illustrated with publicly available microarray data from patients with OPL [3]. The dataset consists of gene expression measurements for 29096 genes and survival outcomes to develop oral cancer on 86 patients. These 86 patients were selected from the 162 patients who were involved in a chemoprevention trial. The number of 35 patients who developed oral cancer during the study period was selected for gene expression profiling. Besides, 51 samples (ad hoc choice) from patients who did not develop OSCC were randomly selected among 106 patients. The median follow-up time was 5 years for this sample. The data analyzed in this study is available from the NIH Gene Expression Omnibus database at www.ncbi.nlm.nih.gov/geo under the accession number GSE26549. Potentially important clinical covariates were age, histology at baseline, deltaNp63, and podoplanin expression at baseline.

### 2.2. Variable Selection via Additive Hazards Model

Regularization techniques are particularly useful for variable selection in high-dimensional setting where the number of variables is much greater than the sample size and have gained increasing popularity. These estimation techniques pose a penalty term on the coefficients in objective function and shrink the estimates of the coefficients towards zero relative to the maximum likelihood estimates. The goal of this shrinkage is to prevent overfitting which arises due to either collinearity of the covariates or high-dimensionality [14]. When the outcome is survival time, the objective function is usually written based on hazards at the failure time and posing penalties on the coefficients can yield sparse models and hence perform simultaneous variable selection and estimation.

Consider a sample of *n* independent observations. Let *T*_*i*_  (*i* = 1,…, *n*) be the time to an event like death from cancer for the *i*th subject and conditionally independent of the censoring time *C*_*i*_, given the p-dimensional possibly time-dependent covariate vector *Z*_*i*_ = (*Z*_*i*1_, *Z*_*i*2_,…, *Z*_*ip*_)^*T*^. Let *X*_*i*_ = min⁡(*T*_*i*_, *C*_*i*_) and Δ_*i*_ = *I*(*T*_*i*_ ≤ *C*_*i*_) for right censored data, where *I*(·) is the indicator function. Then the observed data consists of (*X*_*i*_, Δ_*i*_, *Z*_*i*_). The hazard function of a failure time *T* given a *p*-vector of possibly time-dependent covariates *Z* based on Lin and Ying additive hazards model is as follows:

(1) $$\begin{array}{c} \lambda \left(t;Z\right)=\lambda_{0}\left(t\right)+\beta_{0}^{T}Z, \end{array}$$

where *λ*_0_ is an unknown baseline hazard function which is common to all subjects and *β*_0_ is a *p*-vector of regression coefficients [4, 15]. The pseudo score linear in *β* ∈ *R* ^*p*^ function for Lin and Ying [16] model can be defined as follows:

(2) $$\begin{array}{c} \text{U}\left(\beta \right)=\frac{1}{n}\;\overset{n}{\underset{i=1}{\sum }}\overset{\tau }{\underset{0}{\int }}\left\{Z_{i}-\overset{-}{Z}\right\}\left\{dN_{i}\left(t\right)-Y_{i}\left(t\right)Z_{i}dt\right\}, \end{array}$$

where *N*_*i*_(*t*) = *I*(*X*_*i*_ ≤ *t*, Δ_*i*_ *ε*_*i*_ = *k*) is the observed-failure counting process, *Y*_*i*_(*t*) = *I*(*X*_*i*_ ≤ *t*) is at-risk indicator, $\overset{-}{Z}=\sum_{j=1}^{n}Y_{j}(t)Z_{j}/\sum_{j=1}^{n}Y_{j}(t)$, and *τ* is the maximum follow-up time. Performing some algebraic manipulation results in

(3) $$\begin{array}{c} U\left(\beta \right)=b-V\beta , \end{array}$$

where $b=\left(1/n\right)\sum_{i=1}^{n}\int_{0}^{\tau }\left\{Z_{i}-\overset{-}{Z}\right\}dN_{i}(t)$ and $V=\left(1/n\right)\sum_{i=1}^{n}\int_{0}^{\tau }Y_{i}(t)\left\{\left(Z_{i}-\overset{-}{Z}\right){\left(Z_{i}-\overset{-}{Z}\right)}^{T}\right\}dt$. Because *V* is positive semidefinite, integrating −*U*(*β*) with respect to *β* results in the least squares type loss function as

(4) $$\begin{array}{c} L\left(\beta \right)=\frac{1}{2}\beta^{T}V\beta -b^{T}\beta . \end{array}$$

Then, the penalized estimator $\hat{\beta }$ is a solution to the regularization problem

(5) $$\begin{array}{c} \hat{\beta }=\underset{\beta \in R^{p}}{\arg\min}\left\{Q\left(\beta \right)\equiv L\left(\beta \right)+\overset{p}{\underset{j=1}{\sum }}p_{\lambda }\left(\left|\beta_{j}\right|\right)\right\}, \end{array}$$

where *L*(*β*) is the likelihood of beta for additive model, *p*_*λ*_(*θ*), *θ* ≥ 0 is a penalty function based on the regularization parameter *λ* ≥ 0 and is often rewritten as *p*_*λ*_(·) = *λρ*(·) [4].

The present study considered three commonly used sparse penalty functions which correspond to Lasso, SCAD, and SICA. In this regard, the Lasso uses the *L*_1_-penalty; that is, *ρ*(*θ*) = *θ*. The SCAD penalty is given by the derivative *ρ*_*λ*_′(*θ*) = *I*(*θ* ≤ *λ*) + ((*aλ* − *θ*)_+_/(*a* − 1)*λ*)*I*(*θ* > *λ*) with some *a* > 2 as a shape parameter and SICA penalty takes the form *ρ*(*θ*) = (*a* + 1)*θ*/(*a* + *θ*) and *a* > 0 is a shape parameter. Estimation of $\hat{\beta }$ is then accomplished via the* coordinate descent algorithm *[4].

Selecting the optimal regularization parameter *λ* is conducted through 10-fold cross-validation based on the following cross-validation score function:

(6) $$\begin{array}{c} CV\left(\lambda \right)=\frac{1}{10}\;\overset{10}{\underset{m=1}{\sum }}\;L^{\left(m\right)}\left({\hat{\beta }}^{\left(-m\right)}\left(\lambda \right)\right), \end{array}$$

where *L* ^(*m*)^(·) is the least squares type loss function computed from the *m*th part of the data, and ${\hat{\beta }}^{\left(-m\right)}(\lambda )$ is the estimate from the data with the *m*th part removed [4].

The SCAD and SICA have one additional tuning parameter *a*. For the SCAD penalty, *a* = 3.7 was suggested by Fan and Li [7] from a Bayesian perspective. Selecting *a* for the SICA penalty requires a little more caution, because small values of *a* that often needs to yield a superior theoretical performance may sometimes lead to the computational instability [4]. This study used the method proposed by Lin and Lv [4] to determine *a* in SICA.

### 2.3. Properties of Used Penalties

In spite of choosing the penalty function, the performance of the regularized estimators depends on various factors, such as the dimensionality of the model, the correlation among the variables, and the choice of the regularization parameter. Also, penalization techniques may suffer from some drawbacks.

Although the Lasso method enjoys the advantage of computational simplicity, it suffers from several shortcomings. All coefficients are shrunken toward zero by Lasso. So, the large elements of coefficients tend to be underestimated. The SCAD penalty is proposed to eliminate the bias caused by the Lasso and does not have this drawback. Also, it has been shown to enjoy the oracle property. It means that the resulting estimator performs asymptotically as well as the oracle estimator which knew the true sparse model in advance [4]. SCAD does not require the irrepresentable condition to consistency. In spite of nonconvexity of SCAD, algorithms are available to compute its solution [7, 17]. On the other hand Lv and Fan showed that the regularity conditions needed for the Lasso can be substantially relaxed by using concave penalties due to nonasymptotic weak oracle property [11]. The SICA family proposed in their study has the noticeable feature that it can be used to define a sequence of regularization problems with varying theoretical performance and computational complexity [4].

### 2.4. Predictive Performance

Assessment of the performance of three variable selection methods was performed by using time-dependent receiver operator characteristic (ROC) curves [18] and bootstrap .632+ prediction error curves [19] which were used to evaluate the performance of the models. The prediction error was obtained as squared difference between the true state (0 for being still under risk and 1 if an event of cancer occurred) at time t and predicted survival probability. Lower prediction errors suggest better performance.

### 2.5. Software

Analyses were performed by using the R software programming (http://www.r-project.org) based on a publically available R package which has been provided by Lin and Lv [4] (http://www-scf.usc.edu/~linwei/software.html). In addition, evaluating the predictive performance of used methods was utilized using “pec,” “peperr,” and “survAUC” R packages. Besides, to select the most effective genes, “timeROC” package was utilized.

## 3. Results

Three variable selection techniques of Lasso, SCAD, and SICA were implemented on microarray gene expression data of 86 leukoplakia samples of OPL patients based on additive hazards model. The frequency of occurrences of the genes along with means of coefficients and standard errors over 100 replicates, obtained from three techniques, was shown in Table 1. As shown in Table 1, the number of selected genes by Lasso was greater than SCAD and SICA. The SICA selected only a small set of genes due to the sparseness. In addition, there are seven common genes (7905589, 7908407, 8106919, 8126931, 8161169, 8174970, and 8180388) among three variable selection techniques.

**Figure 1:**
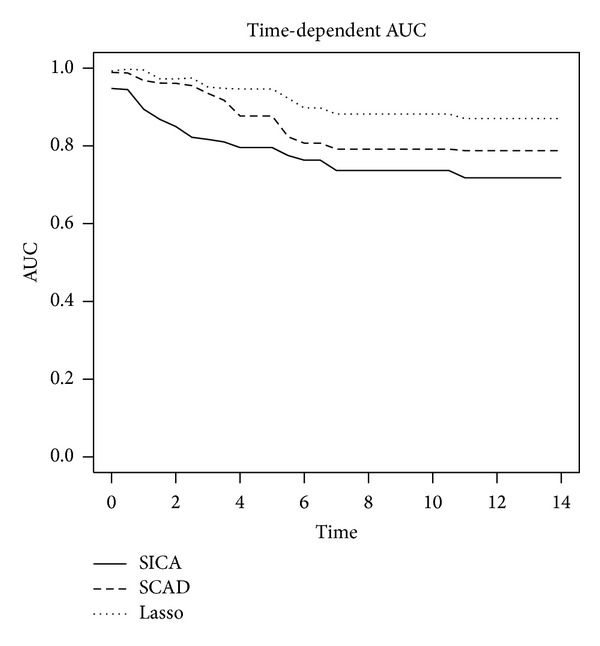
Comparison of predictive performance (area under the ROC curve, over time) for the OPL patients.

In order to assess predictive performance, the median area under ROC curve over time (AUC(*t*)) was computed and plotted based on 69 (~80%) training and 17 (~20%) test sets for each method. Results are shown in Figure 1.

For the follow-up period, the median AUC for Lasso, SCAD, and SICA was 0.95, 0.91, and 0.83, respectively. As can be seen from Figure 1, the predictive performance of the Lasso was superior to SCAD and SICA in this data analysis.

**Figure 2:**
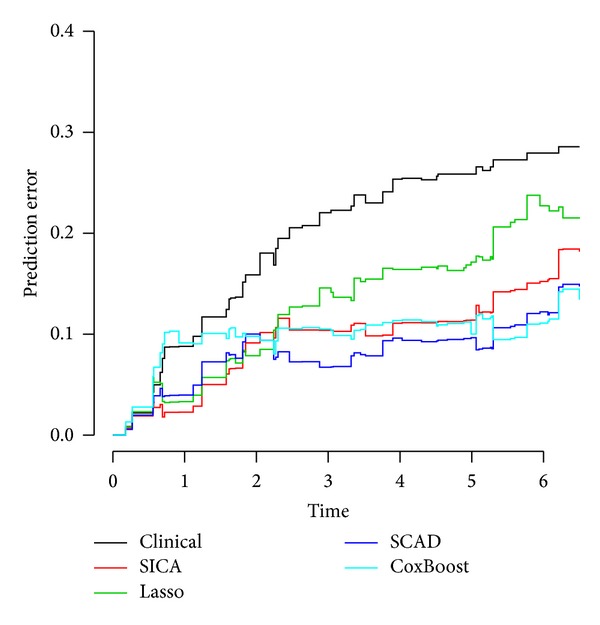
Model comparison using prediction error curves. Clinical model used age, histology, and podoplanin and deltaNp63 expression as predictors. The SICA, SCAD, and Lasso used selected microarray data as well as age, histology, and podoplanin and deltaNp63 expression as predictors.

In addition, to evaluate prediction performance improvement by including selected genes over a purely clinical model, bootstrap .632+ prediction error curves were plotted based on *B* = 100 bootstrap samples drawn without replacement.  Figure 2 shows the results. As shown, including selected microarray features in the models clearly improved prediction performance over the purely clinical model indicating the valuable information containing in the microarray features. Also, it can be seen that the performance of SCAD (bootstrap .632+ prediction error = 0.105) was slightly superior to Lasso (bootstrap .632+ prediction error = 0.124) and SICA (bootstrap .632+ prediction error = 0.132). In order to compare, the prediction error curve of the method utilized by Saintigny et al. [3] to select effective genes named component-wise likelihood-based boosting was also provided in Figure 2. As shown, based on prediction error curve, the performance of SCAD was better compared to component-wise likelihood-based boosting.

Finally, five most effective genes (8126931, 8120206, 7971191, 8161169, and 7897663) were selected using area under ROC curve by means of timeROC package. The median AUC of these genes was 0.825, 0.816, 0.787, 0.786, and 0.702, respectively. Genes 8126931, 8120206, and 7897663 were significant (*P* < 0.001, *P* = 0.01, and *P* < 0.001) by using additive model and the expression of all three genes decreased the survival time.

## 4. Discussion

Due to the low observations for microarray data, only a small number of influential genes can reliably be identified [20]. Therefore, fitting sparse models with only a few nonzero coefficients is interesting [20].

In this study, the performance of three sparse variable selection techniques was investigated based on an additive hazards model for survival data, in the presence of high-dimensional covariates. It was indicated that, based on AUC criterion, Lasso penalty resulted in higher capability of prediction than other methods. The vast majority of genes selected by Lasso approach had frequency greater than 90 percent indicating the stability of the selected gene in this approach. In a study conducted by Ma and Huang [13] the high-dimensional survival time modeled using the additive hazards model. Their results, based on two real data sets, showed appropriate performance of Lasso. Also, in a study conducted by Lin and Lv [4] performance of Lasso in an additive hazards model was reasonable.

Although Lasso performs shrinkage and variable selection simultaneously for better prediction and model interpretation, if there is a group of variables among which the pairwise correlations are very high, then the LASSO tends to arbitrarily select only one variable from the group [21]. This restriction might lead to some problems in the analysis of gene expression data where identification of an entire set of correlated genes may lead to an improved understanding of the biological pathway [5]. Superior performance of the elastic net penalty compared to Lasso was confirmed by Engler and Li based on a Cox proportional hazards model. It is suggested to use the elastic net penalization approach to deal with high-dimensional low-sample size survival data in an additive manner.

Despite the lower number of selected genes, the AUC of the SCAD and SICA was also comparable to the Lasso. On the other hand, based on the prediction error criterion, the SCAD showed minimum value indicating better prediction. Lin and Lv [4] conducted a simulation study to investigate and to compare some sparse variable selection techniques. Their results showed that the performance of SCAD and SICA was good with slight superiority of SICA.

In addition, the selected genes exhibited some consistency across three used methods in terms of selecting common genes. However, variability was observed due to the low-sample size and ultrahigh-dimensionality as well as different penalty functions which were utilized by different techniques [4]. Reducing the dimensionality by some techniques including sure independence screening [22] is suggested before using the regularization methods to gain more stability. Fan and Lv [22] showed that the prediction performance of all methods tends to improve.

Based on the AUC, three genes were diagnosed as the most effective genes on OPL patients' survival. Accordingly, these genes can predict the survival time of the patients with OPL, if they are taken into account simultaneously in an additive hazards model. The expression of genes 8126931, 8120206, and 7897663 can decrease the survival time.

Saintigny et al. [3] conducted a study on the same dataset using a boosting approach which is also a sparse technique. They utilized Cox proportional hazards model and identified a small cluster of genes expression. However, due to the sparseness of utilized techniques, there is only a small overlap with the present study, with only one common microarray feature (8095441). Also the performance of boosting based on AUC (AUC = 0.95) was comparable with three methods used in the present study. However, based on prediction error curve the performance of SCAD was superior. It is suggested that simulation studies are conducted to further assessment of the performance of these techniques as well as different data sets.

One important restriction in the present study was that gene expression data were not identified for a large part of the dataset. Eliminating this part of dataset may result in selection bias in the present study's result. Besides, we could not assess the biological mechanisms and the effect of the selected genes on the pathogenesis or progress of OPL; therefore, it is suggested that the pathogenic effect of reported genes is evaluated in the future studies.

The present study introduced a new set of influential microarray features in predicting OPL patients' survival from a different perspective of the proportional hazards. According to the result of the present study, despite the small number of selected genes three methods of Lasso, SCAD, and SICA showed reasonable performance in additive manner and the selected genes improved prediction performance over a purely clinical model.

The additive risk models and used variable selection approaches provide a useful alternative to existing dimension reduction techniques based on Cox's model for survival data with high-dimensional covariates. The performance of the different models and dimension reduction techniques are data dependent with no method dominating the others [23]. Comprehensive simulation studies and data analysis will be required to draw more definitive conclusions.

## 5. Conclusion

The present study indicated that three feature selection approaches of the Lasso, SCAD, and SICA performed reasonably well in handling high-dimensional time-to-event data corresponding to OPL patients based on additive risk. However, the SICA selected smaller number of genes than the Lasso and SCAD. Furthermore, the selected genes by these methods improved the prediction of purely clinical model and can satisfactorily predict survival.

## Figures and Tables

**Table 1:** Influential genes on OSCC patients' survival based on additive hazards model using Lasso, SCAD and SICA. Values are frequency of occurrences of the genes, means of coefficients (standard errors) over 100 replicates.

Probeset ID	Lasso		SCAD		SICA	
	Frequency	Coefficient (SE)	Frequency	Coefficient (SE)	Frequency	Coefficient (SE)
7897663	100	0.141 (0.006)				
7905589	100	−0.122 (0.032)	97	−0.182 (0.086)	88	−0.324 (0.132)
7908407	100	−0.639 (0.054)	98	−1.239 (0.645)	92	−1.367 (0.460)
7916489	99	−0.012 (0.009)	56	−0.022 (0.021)		
7918825	99	0.112 (0.041)				
7919157	81	0.081 (0.049)				
7922793	99	−0.069 (0.035)	48	−0.012 (0.017)		
7925161					1	−0.002 (0.017)
7946565			44	0.009 (0.010)		
7964627	91	0.102 (0.056)			12	0.030 (0.084)
7965467	99	−0.100 (0.050)	56	−0.034 (0.051)		
7971191	91	0.037 (0.019)	30	0.003 (0.007)		
7978754	100	−0.136 (0.015)	50	−0.005 (0.008)		
7981968	100	−0.086 (0.021)				
7982129	99	−0.012 (0.005)	56	−0.002 (0.003)		
8002247	91	0.100 (0.052)			2	0.002 (0.017)
8018097	100	−0.140 (0.008)	98	−0.060 (0.018)		
8020844	36	−0.011 (0.019)	94	−0.024 (0.012)		
8035398	75	−0.018 (0.012)				
8035829	64	0.018 (0.014)				
8040338	91	−0.016 (0.007)				
8044733	91	−0.039 (0.019)				
8047690			56	0.023 (0.028)		
8048595	91	−0.055 (0.030)	30	−0.005 (0.013)		
8065392	91	−0.168 (0.086)			1	−0.003 (0.029)
8076511	49	0.011 (0.011)				
8075691			26	−0.034 (0.058)		
8093764			56	−0.018 (0.020)		
8095441			56	−0.006 (0.006)		
8103368	100	−0.011 (0.003)	56	−0.009 (0.013)		
8106814	75	0.040 (0.029)	56	0.024 (0.033)		
8106919	81	0.070 (0.046)	97	−0.092 (0.028)	1	0.002 (0.020)
8109828	100	−0.128 (0.003)			47	−0.068 (0.082)
8110880			56	0.047 (0.041)		
8112916			68	0.005 (0.009)		
8120206	91	0.030 (0.015)				
8123338	75	−0.065 (0.045)			1	−0.002 (0.018)
8126931	100	0.157 (0.025)	84	0.022 (0.017)	2	0.002 (0.014)
8138531					90	0.873 (0.338)
8139808	99	−0.059 (0.016)				
8127993			30	−0.008 (0.014)		
8158952			84	0.900 (0.676)		
8161169	100	0.281 (0.029)	97	0.154 (0.041)	70	0.144 (0.175)
8174710	82	−0.023 (0.013)	24	−0.007 (0.012)		
8174970	100	0.360 (0.030)	94	0.060 (0.046)	35	0.101 (0.140)
8180388	100	−0.403 (0.039)	98	−0.139 (0.077)	12	−0.072 (0.199)
